# Determining Real-Time Communication Feasibility in IoT Systems Supported by LoRaWAN

**DOI:** 10.3390/s23094281

**Published:** 2023-04-26

**Authors:** Matias Micheletto, Paula Zabala, Sergio F. Ochoa, Roc Meseguer, Rodrigo Santos

**Affiliations:** 1 CIT Golfo San Jorge Research and Transfer Center (CIT-GSJ), CONICET, Comodoro Rivadavia 9000, Argentina; matias.micheletto@gmail.com; 2 Department of Computer, FCEN-UBA, ICC-CONICET-UBA, Buenos Aires 1428, Argentina; pzabala@dc.uba.ar; 3 Department of Computer Science, Universidad de Chile, Santiago 8370456, Chile; 4 Department of Computer Architecture, Universitat Politècnica de Catalunya, 08034 Barcelona, Spain; meseguer@ac.upc.edu; 5 Department of Electrical Engineering and Computers, UNS, ICIC-CONICET-UNS, Bahia Blanca 8000, Argentina; ierms@uns.edu.ar

**Keywords:** LoRaWAN, real-time communication in IoT systems, feasibility model, heuristic optimization, large wireless sensors networks

## Abstract

LoRaWAN is a long range and low power protocol devised for connecting devices under the Internet of Things (IoT) paradigm. This protocol was not conceived to support real-time message delivery; therefore, it is not always feasible using it to support IoT solutions involving large wireless sensors networks and time constraint messaging, e.g., in early warning systems for natural hazards, remote monitoring of industrial machinery or autonomous control of transportation systems. This paper presents a model that provides certainty, at the design time of IoT systems, about the real-time communication capability of their supporting network. It allows solution designers: (1) to decide if developing or not a real-time IoT solution based on the feasibility of its communication infrastructure, and (2) to improve the communication infrastructure to try making real-time communication feasible using LoRaWAN.

## 1. Introduction

LoRaWAN is one of the leading technologies to support IoT-based solutions, as they can provide connectivity in long distances with a rather low energy demand [1]. It implements a transmission protocol that addresses the physical and link layer, and provides access control based on an ALOHA unslotted protocol.

**Figure 1:**
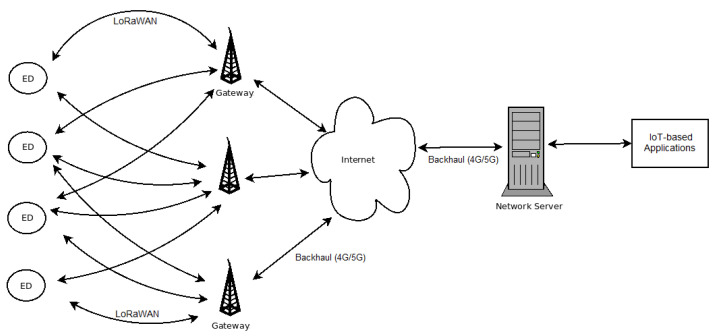
Operation scenario of IoT systems supported by LoRaWAN.

LoRaWAN networks operate in a star topology (Figure 1) that involves end-devices, gateways, a network server and communication links with different purposes. The end-devices (*EDs*), usually sensors and actuators, are connected to one or more gateways. These gateways (*GWs*) concentrate the messages from the *EDs* and forward them to the Network Server (*NS*) through a stable communication link known as backhaul. This backhaul is usually implemented through 4G/5G, wired fiber optic or satellite connections.

In several application domains, like smart cities and industrial IoT, the applications require connecting a large number of *EDs* and operate under time constraints (i.e., performing real-time communication). For instance, in industrial control networks the periodicity of messages delivery can be in the order of tenths per second, which is at least two orders of magnitude smaller than frequencies used in most of IoT applications. Something similar happens in many IoT applications that monitor urban critical infrastructure like airports, traffic light systems or subway systems.

Although LoRaWAN networks work properly when managing messages with low sampling frequencies or large periods, their scalability is not always feasible when thousands of end-devices are connected and time constraints are present for message delivery [2,3]. On the one hand, LoRaWAN packets access the shared channels randomly, producing collisions that affect the network scalability. On the other hand, the real-time communication support provided by LoRaWAN is limited by the duty-cycle restrictions, the overhead protocol operation, and the use of ALOHA as MAC protocol.

In some cases, these limitations can be addressed controlling the density and location of the gateways in the supporting network; i.e., the EDs are located where they are required by the IoT application, but the number and location of the gateways can be established (at the network infrastructure design time) considering the time-constraints communication requirements. Although the system designers can reconfigure the location of the gateways (or add new ones) to try supporting real-time communication, in many cases addressing such a requirement is not feasible.

In order to deal with this situation, the system designers require to count on mechanisms that give them certainty, at the IoT system design time, on the feasibility of performing real-time communication on a particular network infrastructure. Unfortunately, the gateways assignment and location problem in these networks has been proved to be NP-hard [4,5,6,7]. Therefore, it cannot be solved with an exact method; at least not in a reasonable time, since it has exponential characteristics.

In case of IoT communication scenarios supported by LoRaWAN, their design becomes an optimization problem; i.e., it is necessary to determine the minimum number of gateways, and also their locations, to guarantee that all end-devices can transmit their messages on-time to the Network Server through the gateways.

In order to support the communication infrastructure design activity, this paper presents an integer linear programming (ILP) model that determines, at the network design time, the feasibility to perform real-time communication in a particular LoRaWAN network. Moreover, the model allows the designers to establish the number and location of gateways required to reach real-time communication, when that communication type is feasible. The model allows these designers to obtain several alternatives of solution (i.e., the number and location of gateways) using different optimization heuristics.

Section 2 discuses recent proposals to deal with network scalability limitations when time constraint messages must be addressed. The following Section 3 presents background information on LoRaWAN that allows to better understand the communication dynamic of the proposed model. Section 4 presents the design decisions and constraints considered to support real-time communication. Section 5 introduces the proposed model, which allows determining feasibility of real-time communication in a particular LoRaWAN network. Section 6 presents three heuristics to determine the minimum number of gateways to support real-time communication (when it is feasible), and the locations of these nodes. Section 7 describes the experimental evaluation and explains the obtained results. Finally, Section 8 presents the conclusions and future work.

## 2. Related Work

As mentioned before, in several application domains the IoT systems require a communication infrastructure capable of connecting thousands of sensors at the sensing layer, e.g., in smart cities. Many of these nodes must operate under time constraints, therefore, real-time scheduling becomes a key issue to address in large sensors networks. In what follows, we discuss the related work on three aspects highly relevant to deal with the stated challenge: the LoRaWAN limitations in terms of scalability and real-time support, the placement of gateways to allow real-time communication, and the main extensions proposed to LoRaWAN to deal with message synchronization issues.

### 2.1. Real-Time and Scalabitity Support in LoRaWAN

In [3] the authors analyze the limits of LoRaWAN, and identify the scalability problem as one of the main issues caused by the duty-cycle restriction and time needed for message transmission. Aligned with that, in [2] the authors present a survey on challenges for LoRa and LoRaWAN networks, where they identify the link coordination and resource allocation as the main problems to deal with multiple access problem. These authors also indicate that is required a suitable coordination for using the links and allocating end-devices to gateways. In this sense, there are some research works where the gateways location and EDs assigned to them, are treated for the case of wireless sensor networks [8,9]. These proposals consider different issues like energy demand, throughput, and data aggregation.

In  [10] the authors propose a new MAC protocol for LoRa to reach a better scalability. The protocol is backward compatible with LoRaWAN, but incorporate new features (e.g., to allow group acknowledgments), and thus to reduce the required bandwidth and increase the number of end-devices that can be scheduled. This protocol, named DG-LoRa, addresses the network scalability in LoRa, but it does not deal with the gateways allocation problem that is present in LoRaWAN.

### 2.2. Gateways Placement Problem Optimization

In [6] the authors propose a greedy heuristic to minimize the use of gateways in wireless mesh networks. However, when this approach is implemented on LoRaWAN networks, it produces asymmetric loads on the gateways. In [11], the allocation problem is analyzed for the case of LoRaWAN networks. Particularly, end-devices are allocated to gateways using an algorithm based on the Signal-to-Noise Ratio (SNR) and SF range. The objective is to minimize collisions or maximize throughput while saving energy, but without considering time constraints.

In [12] the authors introduced an ILP model to compute an optimal distribution of the end-devices allocated to gateways within a smart-city IoT communication network. The model uses the spreading factor and transmission power together with the geographical distance to build an efficient network. Extensive simulations show that the proposed mechanism improves other allocation algorithms. The authors propose a two-step optimization procedure. First they allocate the end-devices to the gateways based on the SF range. Then, the transmission power used by each end-device is minimized to avoid unnecessary collisions. Although useful, this proposal does not use real-time communication as a factor to allocate end-devices to gateways, therefore, the real-time communication cannot be ensured.

In [13] the authors present an adaptive priority-aware resource allocation mechanism to improve LoRaWAN scalability and energy consumption in a dense IoT scenario. The simulation results show high packet delivery and low delay for high priority applications. However, the approach is not considered real-time because deadlines are not included in the analysis.

### 2.3. LoRaWAN Extensions to Deal with Synchronization Issues

In [14], a synchronization entity is incorporated to the Network Server (NS) to compute a time sharing schedule for the end-devices. New nodes becoming active in the network, when they register with a gateway, are allocated to a particular time slot that is synchronized with certain periodicity to keep clock drifts under control. Although the proposal is interesting, it does not allocate end-devices to gateways, therefore, the it is not clear how this proposal allows LoRaWAN network scale and operate under real-time constraints.

In [15] the authors present an extension to LoRaWAN protocol, called Aggregated Acknowledgment Slotted Scheduling LoRaWAN (A2S2-LoRaWAN), to improve the scalability and reliability of these networks. This protocol contains time-slotted ALOHA-based periodic frame structure, which is supported by aggregated acknowledgment methods for scheduling transmissions. The authors proved an important reduction in the bandwidth requirements, which allows increasing the number of end-devices that can be scheduled to transmit.

In [16] the authors report an extension to the LoRaWAN architecture, and implements a packet-forwarding mechanism between the end-devices of the system. The proposal helps overcome potential infrastructure blackouts after an earthquake; therefore, it provides safety awareness information when it is most needed. The LoRaWAN extension is not oriented to support the real-time communication, but to provide alternative interaction paths between the EDs and the NS.

In [17] a real-time MAC protocol is proposed to provide real-time guarantees in the context of industrial IoT systems. The authors present an heuristic to schedule messages in nodes to facilitate their transmission on-time. Similarly, in [18] the authors propose a new MAC protocol for LoRa to provide real-time guarantees in industrial monitoring and control scenarios. The scheduling is based on the implementation of transmission frames, in which messages are scheduled on a set of logical-index following some heuristic rules. Then, the same authors extended their previous work to address mixed traffic of periodic and aperiodic real-time messages [19]. Both approaches are not based on LoRaWAN but, on LoRa as physical and link layer.

Summarizing, there is a set of previous works that propose modifications to the LoRaWAN medium access control protocol, as the ALOHA mechanism restricts the network scalability when time constraints are present. Next section describes a set of communication design decisions that considers the characteristics of the LoRaWAN protocol and shapes the model proposed to determine feasibility of the real-time communication in these networks.

## 3. LoRaWAN Background

LoRaWAN uses a spread spectrum technique to transmit messages with low power. The technique facilitates the reception of these messages, but at the cost of low bit-rate transmission [20]. As the radio frequency is within the unlicensed spectrum, the duty-cycle (DC) associated to each device is small, being 1% the most used value. These networks specify six spreading factors (SFs), which result orthogonal among them. This allows the EDs to transmit simultaneously using different SFs. Each ED and GW may use different channels; the typical bandwidth (*B*) used by each channel is 125 Khz.

The last parameter to set-up in a LoRaWAN communication is the code rate (CR) that specifies the number of redundant bits to be sent. Typically, this parameter is set to 4/5. With this information it is possible to determine the bit rate (BR) achievable with each possible combination of parameters by computing the Equation (1) [21].

(1) $$BR=SF\frac{B}{2^{SF}}CR$$

From (1) we can see that, while moving up in the SF selection, the BR is almost halved each time, i.e., the time needed to transmit a message is approximately doubled. This expression is provided by manufacturers, and it comes from the modulation used by the radio transducers.

LoRaWAN provides a complete set of working primitives to interconnect EDs with GWs using three different modes of operation: A, B and C. All modes are bi-directional.

In class A, when a message is ready to be transmitted, it wakes up and transmits following an ALOHA based protocol. After an up-link transmission, two short down-link reception windows are open. When the ACK message is received, the end-device goes back to sleep mode.

In class B, the EDs operate in a synchronized fashion with the gateways. The gateway transmits beacon frames at regular intervals of time, and the end-devices use these frames to open reception windows.

Finally, in class C the end-devices are always listening; therefore, the messages can be exchanged at any time. Clearly, this last mode of operation is less efficient in terms of energy consumption, but it has better throughput than the previous ones. All end-devices should operate in class A at the moment of registering with a gateway.

LoRaWAN devices can be tuned in sixteen different channels, and like in the case of the SFs, these channels are orthogonal. This provides a rather large set of combinations (eighty), in which end-devices and gateways may operate simultaneously without producing collisions. The GW devices can listen up to eight channels at the same time [22].

When a node transmits (i.e., an end-device or gateway), its message is listened by every node within the transmission range. To avoid collisions, it is mandatory to guarantee that only one device is accessing the medium at a particular combination of SF and channel.

On the other hand, LoRaWAN introduces a rather long overhead (30 bytes) in each message that affects the real-time communication. Considering the time needed to transmit a byte; e.g., when using SF7 (see Table 1), just the MAC header will require 45 ms. Without sending payload bytes, the 1% DC imposes the minimum period to be 4.5 s. This limits the real-time operation of LoRaWAN, as message requiring shorter update periods are not feasible. This aspect should be considered at the network design time. Table 2 defines the symbols used along the paper.

## 4. Design Decisions and Constraints to Support Real-Time Communication

Recognizing the large diversity of real-time communication scenarios in IoT systems, this proposal is focused on those where the sampling rates are large (i.e., from some seconds to minutes) and LoRaWAN is used as communication protocol. The proposal establishes changes in the configuration of end-devices, gateways and network server, but not in the LoRaWAN protocol; i.e., the proposed modifications do not affect the way in which the schedule of messages is organized in large networks with thousands of nodes. For this, end-devices are operate in class B.

The proposal also includes a time sharing mechanism, in which end-devices transmit their messages to a particular gateway at predefined instants using a specific spreading factor and channel. This communication dynamic is inspired in the proposal presented in [14]. As real-time operation should be predictable at the network design time, we have assumed the following characteristics for the communication model:

- Time is considered to be discrete and the time unit is the slot. Events are synchronized with the beginning of the slots.
- For simplicity, it is assumed that when changing from $SF_{i}$ to $SF_{i+1}$, the number of slots required to transmit the message is duplicated.
- It is assumed that the transmission range is doubled with each increment in the SF.
- Each $ED_{i}$ transmits a sequence of periodic messages characterized by $\left(Z_{i},T_{i},D_{i}\right)$. It is assumed $T_{i}=D_{i}$ and $\forall i\;Z_{i}=Z$. All time units are expressed in slots.
- The time required to transmit one message, with $SF_{7},B=125$ KHZ and CR=1, is the time unit or slot.
- All end-devices used the same transmission power.
- A non-preemptive earliest deadline first policy is adopted for end-devices transmission scheduling [23].

Assuming these design decisions, next subsections describe the communication dynamic and constraints considered to support real-time messaging.

### 4.1. Communication Dynamic

When an end-device (ED) becomes active, it has to register within the network server by selecting for this the best possible gateway through which it will transmit messages to that server. However, when dealing with real-time messages, the common criteria of choosing the gateway with the best RSSI is not necessarily used, as end-devices should be distributed to comply with time restrictions. Thus, in some cases a gateway with lower RSSI will eventually be chosen.

**Figure 2:**
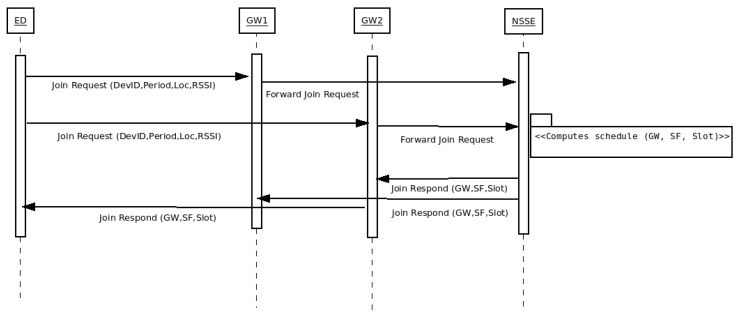
Sequence Diagram for the Join Procedure of an ED.

Typically, an ED begins its registration sending an uplink message to all the gateways within communication range, indicating its location, device identification (DevId), message period and RSSI. The gateways (GWs) forward the messages to the network server where the Network Synchronization and Scheduling Entity (NSSE) is located [14]. Based on the information provided by the ED, the NSSE defines the gateway, spreading factor and time slot in which the end-device should transmit. Once this information is received by the ED, it can begin its participation in the network. Figure 2 shows the sequence diagram of this joint procedure.

### 4.2. Constraints to Support Real-Time Messaging

Let Γ be a set of real-time flows $F_{i}$. Each flow is associated to only one $ED_{i}$ and described as a stream of periodic messages with period $T_{i}$, size in bytes $Z_{i}$ and deadline $D_{i}$. In each period, a new *instance* of the message is generated for its transmission, which should be sent before its deadline $D_{i}$. For simplicity, it is assumed that the absolute deadline is equal to the period.

As previously explained, the time needed to transmit a message depends on three factors: the spreading factor SF, the bandwidth *B* and the code rate CR. Considering these elements, a real-time flow $F_{i}$ will require a transmission time given by $C_{i}\left(Z_{i},SF_{i},B_{i},CR_{i}\right)$  (2).

(2) $$C_{i}=\frac{8Z_{i}}{BR_{i}}$$

where the subindex identifies the $ED_{i}$ that transmits the flow. On top of this, the transmission power $TP_{i}$ of the ED can be used to limit the range to which it can transmit. $BR_{i}$ comes from Equation (1) and states the bit-rate at which the $ED_{i}$ transmits and receives information depending on the radio parameters set-up. The time demanded to transmit $Z_{i}$ bytes is simply the product of $BR_{i}$ and eight times $Z_{i}$, as the number of bits per byte is 8.

**Lemma** **1**. *A periodic real-time flow $F_{i}$ is not feasible if there is not a configuration setup that satisfies:*

$$\frac{C_{i}\left(Z_{i},SF_{i},B_{i},CR_{i}\right)}{T_{i}}\leq 0.01$$

**Proof.**  LoRa in general, and LoRaWAN in particular, define the duty-cycle or maximum allowable percentage of transmission time to be 1%. This hard bound should not be over-passed for the system to be feasible. As the transmission time is a function of the message length in bytes and the radio transducer set-up, if the duty-cycle bound cannot be met, the real-time flow is not feasible.    □

While the period depends on the application needs, the time required to transmit the information depends on the ED configuration setup. There may be several possible combinations of SF and *B* that satisfied the DC, but it may be the case that it is not possible to comply with this restriction. In that case, the only possibility (if the application allows it) is to change the periodicity of the ED, or assume that real-time communication is not feasible for that node.

On the other side, the gateways should listen to end-devices in an ordered way, as to not collide messages. The number of messages a gateway can process is limited by the periods of the devices connected to it, and the SF that is used.

Then, the real-time scheduling problem can be analyzed with the techniques that allows allocating tasks to processors [24,25,26], as messages should complete their transmissions on time and accessing a unique gateway that forwards them. Assuming the gateway operates in just one channel, we can consider each SF as an independent system in which messages may be scheduled until reaching 100% utilization factor.

Let us suppose the set of messages $M_{SF_{k}}=\left\{\left(C_{i},T_{i}\right)\right\}$ that the gateway transmits to the network server in $SF_{k}$. Once an ED begins transmission, it cannot be preempted as the protocol has a large overhead.

**Lemma** **2**. *A gateway $GW_{j}$ is feasible when its incoming messages are scheduled by a non-preemptive earliest deadline first policy if the following conditions are satisfied:*

(3) $$\begin{array}{cc} \forall i\;\;\forall k & \max_{i}<C_{ik}<\min_{i}T_{i} \end{array}$$

(4) $$\begin{array}{cc} \forall SF_{k} & \sum_{\forall M_{SF_{k}}}\frac{C_{ik}}{T_{i}-\max_{i}C_{i}k}\leq 1 \end{array}$$

*where $C_{ik}$ stands for the maximum transmission time of any message allocated to the GW using $SF_{k}$. It must be noted that as it was assumed that all messages within the system have the same length $\max_{i}C_{ik}=C_{ik}$. For simplicity the k subindex can be dropped.*

**Proof**. Gateways listen to the six SF simultaneously. Nevertheless the SF are orthogonal among them. Thus, the gateway can be seen as six different sinks where messages are sent. Each sink has a maximum processing capacity that should be respected to guarantee that all messages are received on time. Trivially this upper bound is 1, this indicates the gateway can handle all the messages arriving with that SF. However, as the ED transmission is not preemptable (it means that once an ED begins a transmission it is not interrupted until the last byte has been sent), the capacity is reduced to consider the time an ED is blocked from transmission as proved in [23].    □

If the utilization factor is greater than one, for any of the SFs, the gateway will not be able to forward the messages [23]. To avoid this, a careful time scheduling of messages is necessary, together with a proper allocation of end-devices to gateways.

Even if all nodes within the network comply with the condition stated in Lemma 1, we still need to find a feasible schedule. For this, each end-device should be associated to a gateway with a proper SF and channel. As all gateways within the transmission range of an end-device may listen to it (to avoid collisions), at each time only one node should be transmitting in a particular SF and channel.

## 5. Real-Time Communication Feasibility Model

The feasibility model considers a set of end-devices that have messages to be scheduled. Each end-device is allocated to one gateway using a specific channel and SF. End-devices and gateways must satisfy the Lemmas 1 and 2 respectively. This allocation is an optimization problem that considers the design decisions and constraints stated in the previous section. In order to address it, we defined an Integer Linear Programming (ILP) model that describes the particular characteristics of LoRaWAN networks, in which end-devices and gateways operate using different SF and channels, and have duty-cycle and time restrictions.

**Figure 3:**
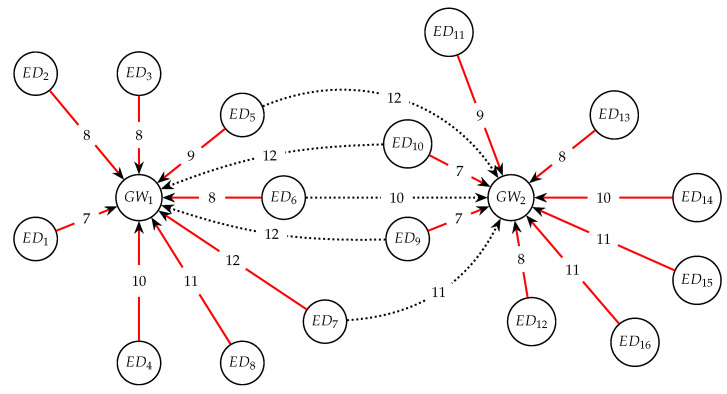
Gateway rooted tree graph description.

Figure 3 shows a clustering example where sixteen end-devices are grouped in two cluster trees. As can be seen, $ED_{5}$, $ED_{6}$, $ED_{7}$, $ED_{9}$ and $ED_{10}$ impact with different SF on both $GW_{1}$ and $GW_{2}$. However, only one of them is used as gateway. The dotted lines indicate the not used links. The allocation procedure of EDs to GWs proposed in what follows, selects for each ED a unique SF and channel, in such a way that only one gateway is used. Particularly, in some deployment scenarios an ED can reach more than one GW. If this occurs, these gateways will forward the message to the Network Server, and it decides on such allocation.

In the ILP model, the sets (5) and (6) define the location of every ED and GW in the map. As shown in (7), it is possible to calculate the Euclidean distance between any two elements in both sets.

(5) $$E=\{ED{\left(e_{x},e_{y}\right)}_{i}\}$$

(6) $$G=\{GW{\left(g_{x},g_{y}\right)}_{j}\}$$

(7) $$dist\left(GW_{j},ED_{i}\right)=\sqrt{{\left(g_{jx}-e_{ix}\right)}^{2}+{\left(g_{jy}-e_{iy}\right)}^{2}}$$

Each GW can be considered as the center of six circles with different radios; each one representing a different communication threshold. The EDs located within each circle may reach the GW using the different SFs. Moving away from the center increases the SF needed to send messages. The radios are problem dependent as they vary with the kind of environment in which the network is deployed. In this case, we assume a set of values measured experimentally in [27]. In (8) the distances associated to each SF are shown.

(8) $$R=\{R_{k}\;\;|\;\;k=7,\ldots ,12,\;\left(7,62.5\right),\left(8,125\right),\left(9,250\right),\left(10,500\right),\left(11,1000\right),\left(12,2000\right)\}$$

There are sixteen possible channels within LoRaWAN to be used by gateways and end-devices. These channels are orthogonal; therefore, neighbor EDs may operate in different channels using the same SF without interfering each other. Equation (9) indicates the set of channels that can be used.

(9) C={ch|ch=0,…,15}

Each $ED_{i}$ has to send a message every $T_{i}$. All messages have the same length, but the transmission time depends on the SF that is used. Equation (10) computes the *utilization factor demand* or *bandwidth demand* that the $ED_{i}$ places on a GW when connected at SF equal to *k*.

(10) $$u_{ik}=\frac{2^{k-7}}{T_{i}-2^{k-7}}$$

The fact of scheduling messages with a non-preemptive earliest deadline first policy is considered by reducing the message period $T_{i}$ [23]. As all messages transmitted to a GW should be received on time, each GW can schedule up to a maximum capacity for each SF equal to 1, in compliance with Lemma 2.

When an ED is listened by more than one GW in a certain SF, its transmission impacts on all of them. If the capacity of the GW is exceeded (it is over 1), a different GW should be used in a different channel to avoid interferences. Considering that, the $ED_{i}$ to $GW_{j}$ allocation problem can be modeled using the following binary variables, objective function and constraints.

(11) $$\begin{array}{c} \begin{array}{cc} w_{jch} & =\left\{\begin{array}{cc} 1 & \mathrm{if}\;GW_{j}\;\mathrm{uses}\;\mathrm{channel}\;ch\\ 0 & \mathrm{otherwise} \end{array}\right. \end{array} \end{array}$$

(12) $$\begin{array}{c} \begin{array}{cc} x_{ij} & =\left\{\begin{array}{cc} 1 & \mathrm{if}\;GW_{j}\;\mathrm{listens}\;\mathrm{to}\;ED_{i}\\ 0 & \mathrm{otherwise} \end{array}\right. \end{array} \end{array}$$

(13) $$\begin{array}{c} \begin{array}{cc} y_{ikch} & =\left\{\begin{array}{cc} 1 & \mathrm{if}\;ED_{i}\;\mathrm{is}\;\mathrm{active}\;\mathrm{in}\;SF_{k}\;\mathrm{and}\;\mathrm{channel}\;ch\\ 0 & \mathrm{otherwise} \end{array}\right. \end{array} \end{array}$$

We consider that a gateway and an end-device listen to each other when they operate within the same channel, and the distance is covered by the SF used. In this scenario, the objective function of the model is to minimize the number of gateways required to address time constraints in the message delivery, as shown in (14):

(14) $$\begin{array}{cc} \min\; & \sum_{j}\sum_{c}w_{jc} \end{array}$$

subject to

(15) $$\begin{array}{ccc} & \sum_{c}w_{jch}\leq 1 & \;\;\;\;\;\;\;\;\;\;\;\;\;\;\forall j\in G \end{array}$$

(16) $$\begin{array}{ccc} & \sum_{j\in G}x_{ij}\geq 1 & \;\;\;\;\;\;\;\;\;\;\;\;\;\;\forall i\in E \end{array}$$

(17) $$\begin{array}{ccc} & \sum_{c}\sum_{k}y_{ikch}=1 & \;\;\;\;\;\;\;\;\;\;\;\;\;\forall i\in E \end{array}$$

(18) $$\begin{array}{ccc} & x_{ij}+\sum_{k}y_{ikch}\leq w_{jc}+1 & \;\;\;\forall j\in G\;\forall ch\;\in C\;\forall i\in E \end{array}$$

(19) $$\begin{array}{ccc} & w_{jch}+\sum_{k}y_{ikch}\leq x_{ij}+1 & \;\;\;\forall i\in E\;\forall j\in G\;\forall ch\in C \end{array}$$

(20) $$\begin{array}{ccc} & \sum_{i\in E}y_{ikch}*u_{ik}\leq 1+M\left(1-w_{jch}\right) & \;\;\;\;\forall j\in G\;\forall k\;\forall ch\; \end{array}$$

(21) $$\begin{array}{ccc} & x_{ij}+\sum_{k}\sum_{c}y_{ikc}\leq 1 & \;\;\;\;\;\;\;\;\;\forall i\in E\;\forall j\in G \end{array}$$

The first constraint (15) indicates that at each GW, if active, it uses only one channel c∈C. Constraint (16) indicates that each ED is listened at least by one GW. Restriction (17) makes each ED to use only one channel c∈C and one $SF_{k}$. In constraints (18) and (19) the sum is made over all the $SF_{k}$, such that the ED is listened by $GW_{j}$. Constraint (18) ensure that if $GW_{j}$ is chosen to listen to $ED_{i}$, then they work on the same channel, while constraint (19) state that if $GW_{j}$ and $ED_{i}$ work on the same channel with an appropriate $SF_{k}$, they are listening to each other. Constraints (20) limit the utilization factor for each active GW in each SF; for this all the utilization factors of the ED listened by $GW_{j}$ are added for each SF, and should not be greater than one. The last constraint (21) is an exclusion restriction, as it forces each ED not to use a $SF_{k}$ lower than the minimum with which it is listened by $GW_{j}$. This ILP model was programmed in CPLEX v20.1.0 with the default parameters (https://www.ibm.com/docs/en/icos/20.1.0, accessed on 19 April 2023)  

## 6. Dealing with the Allocation Problem in LoRaWAN

The allocation of end-devices to gateways is conditioned by the messages’ periods and the Euclidean distance between both nodes that limits the SF to use. End-devices can operate in different SF, but usually only some of them can be used to reach the assigned gateway. As it is shown in Lemma 1, time restrictions (particularly, messages’ periods and DC) may limit the use of higher SFs. As transmission time doubles when the SF is incremented in one unit, an end-device with period 100 and a message of one unit to transmit, may use $SF_{7}$ complying with the DC restriction. However, it may not use $SF_{8}$ as it would violate the DC.

The distance to a possible gateway is the other factor that conditions the allocation process. Again, an end-device may connect to a close gateway using $SF_{7}$, but not to another one with a higher SF, since it violates the DC restriction. If the ED can use a higher SF, then its messages will reach several gateways, although only one of them would forward the message to the Network Server. Therefore, using one or another SF is not the same, as the eventual interference should be considered. The message period and distance to the potential gateways are the variables that allow determining the feasibility to link an end-device to a gateway.

The end-devices distribution within a certain area is another key design aspect to consider when determining the number of gateways required to schedule all the messages. If end-devices are distant among them, probably more gateways would be required to cover the area where the network is deployed, even if it is possible to use larger SF and the gateway utilization factor (*U*) is low.

On the other side, when the concentration of nodes is high in a reduced area, one gateway may be enough to schedule all the messages. However, if the number of end-devices is too large, such that the *U* of the gateway is over 1, more gateways operating in different channels would be necessary to handle all the traffic. The node density is then another important issue at the moment of deploying gateways.

Eventually, the system would not be feasible if end-devices and messages are grouped in a reduced area, in a way that the available gateways are not enough to handle the traffic. Next we describe the optimization heuristic proposed to solve the stated allocation problem. We also present the algorithms for positioning gateways, and connecting them to end-devices.

### 6.1. Resource Allocation Optimization Algorithm

The periods of messages are part of the application requirements, but the transmission time of the messages is a function of the selected SF. The application of Lemma 1 determines a set of possible SF for each ED. The heuristic allocates each ED to only one GW, sets the channel, the SF and the instant at which the ED is allowed to transmit complying with Lemma 2. If more than one time slot is allocated because a SF>7 is selected, then the transmission is not preemptible. In case the ED reaches two gateways operating in the same channel, both gateways should mark the slot as temporarily allocated to that end-device, although only one of them will finally forward the message to the network server.

There is a trade-off between complying deadlines and duty-cycle, and minimizing the number of gateways to be deployed in the network. In order to satisfy deadlines and keep the duty-cycle, it is necessary to keep the SF in the lower range; however, for reducing the number of gateways it is better to use higher SF. The methods proposed in this work reach a compromise between both requirements.

Algorithm 1 presents the pseudo-code of the process followed to optimize the allocation of end-devices to gateways. Based on the gateway positioning method that is used, the algorithm iterates until a feasible solution is obtained, the number of iterations is completed, or the time-out limit is reached.

The algorithm has four configuration parameters: (1) the gateway positioning method to be used, (2) the progress threshold, (3) the stagnation threshold and (4) the maximum spreading factor allowed for gateways, line (1).

Three different methods were implemented to position the gateways: *greedy*, *random* and *pseudo-springs;* lines (8), (11) and (13) show the call to these methods respectively. The greedy allocation method runs in one step, until all the end-devices are assigned to one gateway. The last two methods iterate several times, incorporating gateways as needed until full coverage is reached, i.e., when there are no more disconnected end-devices left.

Initialising the method with a high number of gateways speeds up the process, but it does not guarantee that the solution will be optimal. To improve the coverage, the gateways are moved following different positioning strategies, but if after a few iterations the coverage does not improve anymore, new gateways should be added to the network, as indicated in lines (14) to (20). For this, two parameters are used to control the timing to add new gateways, the progressThreshold and stagnationThreshold

The progressThreshold (shown in (22)) contributes in determining if the newly computed solution is considered or not an improvement respect to the previous one. In this proposal, the number of new connected end-devices, after each step, is used as an indicator. Moreover, the threshold value is selected proportional to the total number of EDs in the network, divided by a factor of 1000. For example, in a network with ten thousands end-devices, eleven new EDs should be connected after each optimization step to be considered a significant progress. After a certain number of steps without improvement or progress, a new gateway will be added. This is where the stagnationThreshold parameter (23) comes in. By trial and error, it was determined that a value of ten allows obtaining acceptable results for most cases. To achieve better results, it is recommended to fine-tune these two parameters.

(22) $$\begin{array}{c} progressThreshold=1+\frac{\mathrm{Number}\;\mathrm{of}\;\mathrm{ED}\;}{1000} \end{array}$$

(23) $$\begin{array}{c} stagnationThreshold=10 \end{array}$$

**Algorithm 1:** Resource allocation optimization algorithm

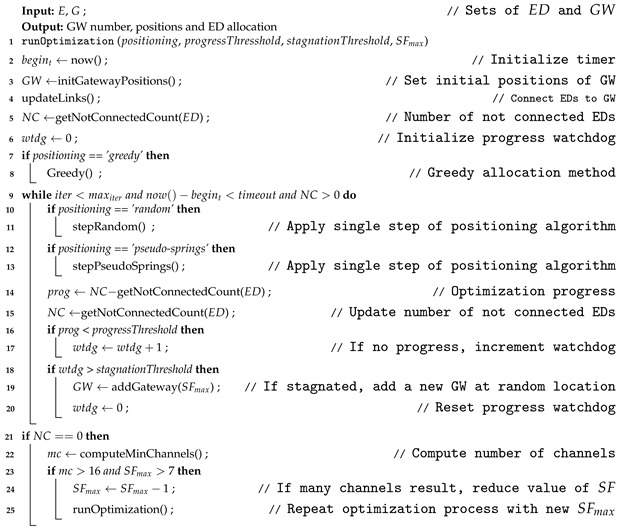



### 6.2. Algorithm to Assign End-Devices to Gateways

Greedy, random and pseudo-springs methods use the same algorithm (Algorithm 2) to connect end-devices to gateways. What is different among them is the strategy used to position the gateways into the network.

The function updateLinks sorts the gateways for each end-device in ascending order considering the euclidean distance, line (1). After that, the reachable gateway with the lowest SF and enough available utilization factor, *U*, is selected, lines (8) to (11). Several gateways may be impacted by one end-device with a certain $SF_{k}$; in that case, the time schedule should consider the transmission of that end-device for all the involved gateways. If one gateway cannot handle such an end-device, then it should use a different channel.  
**Algorithm 2:** Algorithm to connect end-devices to gateways
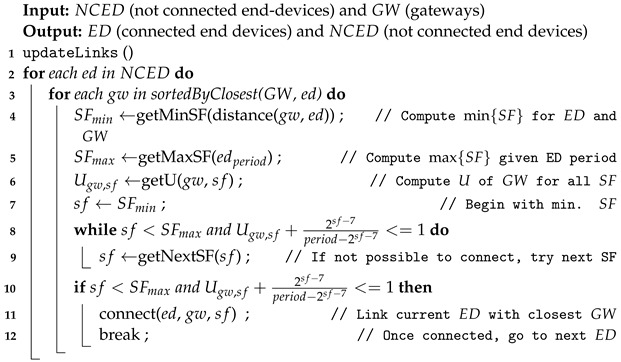


    As mentioned before, the use of channels is limited to 16 in the LoRa physical layer [21], and the transmission distance depends on the SF and power used. As we suppose that every end-device uses the same transmission power, the distance is a function of the SF.

If two nodes interfere with each other in such a way that they cannot be scheduled, one of them should change its transmission channel. If the number of channels used is over 16, then the system is not feasible in that configuration. In that case, the maximum SF used should be reduced, and probably new gateways would be deployed as the transmission distances of the end-devices are reduced too. This process is repeated until all end-devices are allocated to gateways and the total number of channels used is under 16.

To determine the minimum number of channels required to support the operation of the network gateways, a chromatic number algorithm is applied to the graph of gateways. This graph is built connecting gateways with edges when the range of two different gateways produce an overlapping region. Following this strategy, it may happen that there is no possible solution, as the time demand of end-devices can exceed the capacity of the gateways and the possibility of channel differentiation is not available anymore.

### 6.3. Positioning Gateways Using Different Approaches

Once explained the algorithms for gateways assignment and the optimization, in this section we present the algorithms used to position the gateways. Particularly, we show three alternatives of positioning approaches: greedy, random and pseudo-springs. However, more approaches can be added to the model, and used with the Algorithms 1 and 2.

#### 6.3.1. Positioning Algorithm Using a Greedy Approach

This gateways positioning method is presented in Algorithm 3. It considers that every end-device in the system may be turned into a gateway. For this, an adjacency matrix *A* is built, in which each element $a_{ij}$ represents the lowest SF with which the end-devices *i* and *j* can connect, based on the distance and period of the messages to be transmitted.

Once the matrix is computed, the end-device with the highest adjacency degree (i.e., the sum of all elements in a row) is selected as gateway. After this, the utilization factor for each SF is computed before adding the end-device to the selected gateway. Then, the elements in that row and the corresponding elements in the different columns are turned to zero. Therefore, the adjacency degree is computed again for all the end-devices still not allocated, and the process is repeated until all end-devices has been connected to a gateway, or what is the same, all the elements in the adjacency matrix are zero.

Finally, the number of channels is checked using the Algorithm 1, and in case it is greater than sixteen, the maximum allowed SF is decreased and the whole process is run again.
**Algorithm 3:** Gateways greedy positioning algorithm
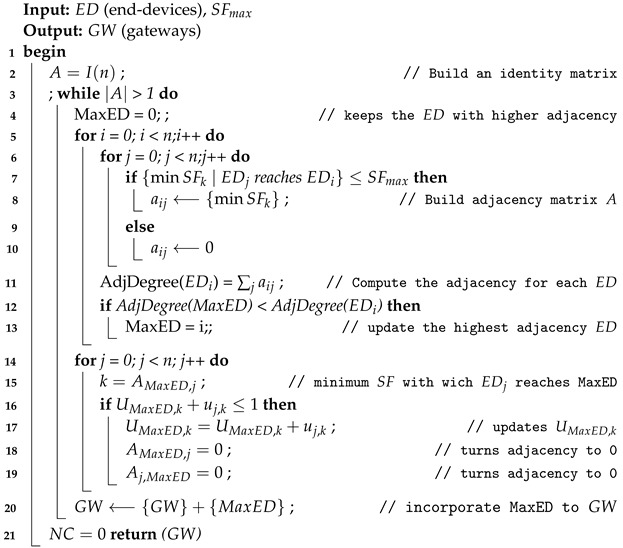


#### 6.3.2. Positioning Algorithm Using a Random Distribution

This gateways positioning strategy is rather simple. Considering that the distribution of end-devices is random, the gateways locations are selected using a uniform random distribution. Particularly, at each step, a new GW distribution is generated. If the percentage of coverage achieved decreases, then the positions are reverted to the previous step. If after a series of attempts no improvement is achieved, then a new GW is added. The number of steps to perform before adding a GW is a configurable parameter.

Algorithm 4 shows the procedure of a single step, which will be performed iteratively. This method improves rapidly the distributions of gateways at the beginning, but after a few iterations it begins to stagnate, as the probability of finding a better GW distribution becomes lower. Although the final solution depends on the number of gateways and the dimensions of the network area, this method serves as reference to evaluate the suitability of other positioning techniques.
**Algorithm 4:** Gateway random positioning algorithm
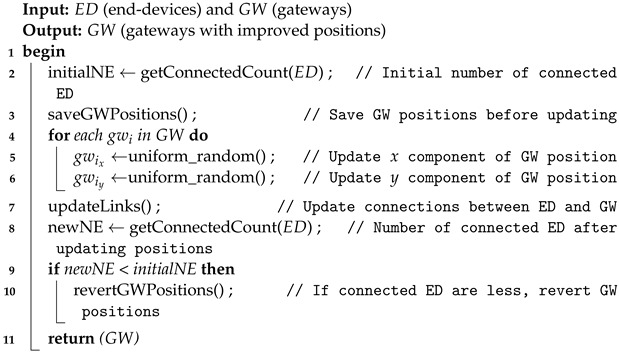


#### 6.3.3. Positioning Algorithm Using a Pseudo-Spring Model

In this case, the objective function consists in determining the minimum number of GW to schedule all messages from end-devices. As stated above, it requires to find a method that efficiently positions the gateways in appropriate places. Taking into account that each ED in the network is more likely to be connected to a nearby gateway, the equations of a dynamic system are proposed to give mobility to the gateways by simulating attraction forces acting on them [28].

In this positioning approach, the initial state or positions are randomly selected. Eventually, they can be man-placed with certain knowledge of the network to schedule.

On the one hand, disconnected *ED*s attract the nearest *GW* as to be able to connect to them when they are in a valid transmission range. On the other hand, the connected EDs attract the GW to which they are currently connected to, in order to position them in what would be the center of mass of that cloud (i.e., the subset of *ED*s). The latter force should be usually weaker than the former, and it allows balancing the distribution of GW in the network space.

Then, the position $x_{g}$ of a certain GW is updated according to its velocity $\dot{x_{g}}$, which follows the Equation (24), where $ED_{c}$ is the set of connected EDs to the current GW, $ED_{n}$ is the set of closest non-connected EDs, $y_{i}$ are the ED positions, and the constants $k_{n}$ and $k_{c}$ are responsible of regulating the strength of the forces acting on the GW. If these constants are defined as the inverse of the numbers of connected or not-connected ED, then the total attraction force (or velocity) is averaged, keeping them in reasonable values. To avoid GW reaching high speeds, a *clamp* function is applied to limit the value to which the positions are updated.

(24) $$\dot{x_{g}}=\sum_{i\in ED_{n}}k_{n}\cdot \left(y_{i}-x_{g}\right)+\sum_{i\in ED_{c}}k_{c}\cdot \left(y_{i}-x_{g}\right)$$

This method allows us to experiment with more complex equations, for instance, by introducing physical variables such as mass or inertia, to change or eventually improve the behavior of the system. The Algorithm 5 shows the procedure for a single step of the optimization process that uses the pseudo-spring model [28].
**Algorithm 5:** Gateway pseudo-spring positioning algorithm
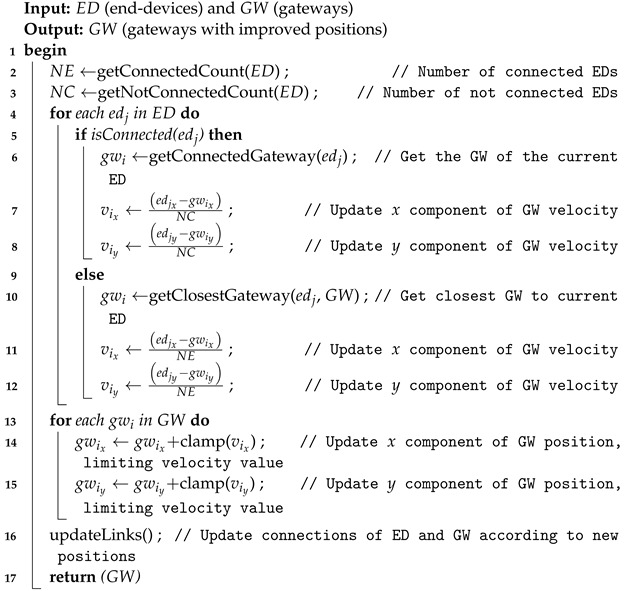


## 7. Simulations and Experimental Results

The suitability of the proposed model was evaluated through various experiments, in which we used the three gateways positioning approaches. The results were compared for synthetic sets of problems with different characteristics.

The experiments were carried out on a server with 2 x Intel(R) Xeon(R) Silver 4210R CPU (10 cores/20 threads) @ 2.40 GHz, 768 RAM DDR4-2400 ECC LRDIMM, using a virtual machine with 10 cores, 128 GB RAM, and Debian 11 Linux/GNU distribution. The implementations were written in C++, and the source code is publicly available in an online repository (https://github.com/matiasmicheletto/realtime-lorawan-sirmulator, accessed on 19 April 2023). A web application was also developed and put it available (http://www.ingelec.uns.edu.ar/rts/LoRaAllocSim, accessed on 19 April 2023), as a way to facilitate the network configuration, simulation and visualization of results. This application uses a WebAssembly (https://webassembly.org/, accessed on 19 April 2023) implementation of the heuristic, compiled from C++ with the Emscripten software (https://emscripten.org/, accessed on 19 April 2023), and a ReactJS (https://reactjs.org/, accessed on 19 April 2023) based GUI.

### 7.1. Experiments Setup

The experiments were performed on synthetic cases, specially conceived to evaluate the behavior of the model in different scenarios and using different positioning approaches. Two kind of experiments were designed to evaluate the performance of the proposed allocation and scheduling methods. The first set of experiments evaluated the objective function considering variability of nodes density and time demand, according to the following:

- Three square maps with sides of 100 m, 1000 m and 2000 m.
- Three time configuration demands: hard, medium and soft.
- Two end-devices distribution: uniform and clouds.
- Five sets of end-devices with 1000, 2000, 5000, 10,000, 20,000 elements each for each map/time/distribution configuration.

The second set of experiments considered constant density of nodes and memory footprint; particularly:

- Three square maps: 500 m, 1000 m and 2000 m side of the square.
- Medium real-time demand.

In the first experiments, 900 instances were evaluated with the three positioning approaches presented in Section 6.3. We compare the performance of these approaches for the two end-devices distribution approaches, in order to solve the problem on two main aspects: *number of necessary gateways, time needed to find a solution.* In total 2700 runs were performed.

The second experiment was designed to determine if the optimization result is better when considering large areas, although it requires more time, or if solving consecutive smaller areas in less time would provide a good enough solution.

### 7.2. The First Set of Experiments: Details and Results

The size of the network area is a key aspect when configuring a LoRaWAN network. LoRa technology can reach long distances at the cost of using higher SF. As explained in Section 3, it increases the transmission time. Both facts become a trade-off for the minimization of gateways. On the one hand, in a sparse area (where end-devices are distant among them), the network will require a relative high number of gateways with low utilization factors, and probably not using many channels. However, when the node density is increased, the gateways reach higher utilization factors, and in small areas probably several channels should be used to accommodate all the transmissions. Next sections explain the distribution of end-devices in the deploying area, the time demand requirements and the obtained results.

#### 7.2.1. Distribution of End-Devices in the Deploying Area

Two different random distributions were simulated. First, end-devices were uniformly distributed in the target area. In this case, the gateways were also located following a uniform distribution, trying to aggregate as much end-devices as possible in each gateway. The number of gateways can be previously approximated, if the density and time demands are known.

The second distribution is considered scenarios where end-devices are concentrated in regions (known as clouds). This may happen for example in residential areas, where end-devices are deployed within buildings and not in parks or open places. The same happens in urban areas, where the downtown usually concentrates a higher density of nodes. The cloud distribution reflects this situation more appropriately than the uniform one.

#### 7.2.2. Time Demand Requirements

In Section 3 we explained the transmission times for the different SF. Considering a payload of ten bytes for all messages, the time needed to send a message, using $SF_{7}$, is 60 ms. This forces the shorter period to be higher or equal to 6 s.

Let us assume that the slot duration of the system is equal to the time needed by an end-device (using $SF_{7}$) to send a message, which is 60 ms. The periods selected for the evaluation of the proposal are in line with what is expected from monitoring applications in smart environments. For example, the shortest period considered is 19.2 s. This can be associated to a sensor requiring three updates per minute. The longest period instead is 16 minutes or roughly four times per hour. Monitoring weather, traffic, pollution or elderly people are samples of applications that can operate within these sensor update rates easily.

The DC restriction imposes limits on the SF used to transmit, according to distances and messages periods. When the end-devices have shorter periods, the possibility of using higher SF is reduced, and therefore, more gateways are necessary as the distances covered by lower SF are shorter. In some cases, when the number of end-devices is high, the real-time scheduling is not possible. The three kind of constraints considered in the simulations show the way in which the several approaches solve the allocation and scheduling of the systems.

As mentioned before, the time unit for each slot is 60 ms, which is the time needed to transmit a LoRaWAN message with ten bytes payload at $SF_{7}$. Systems having soft time constraints use the following periods: 3200, 4000, 8000 and 16,000 slots. In systems that use medium time constraints the periods are: 1600, 2000, 4000 and 8000 slots. Finally, in case of hard time constraints the periods are: 320, 400, 800 and 1600 slots.

Considering the DC restriction, in hard time constraints no end-device may transmit using $SF_{12}$, while in the medium ones only those end-devices with period 4000 and 8000 can use $SF_{12}$. In the case of soft demands, all end-devices can use the maximum SF. Within each time constraint group, all periods have equal probability, i.e., 25% of the end-devices have each period.

#### 7.2.3. Results of the First Set of Experiments

**Figure 4:**
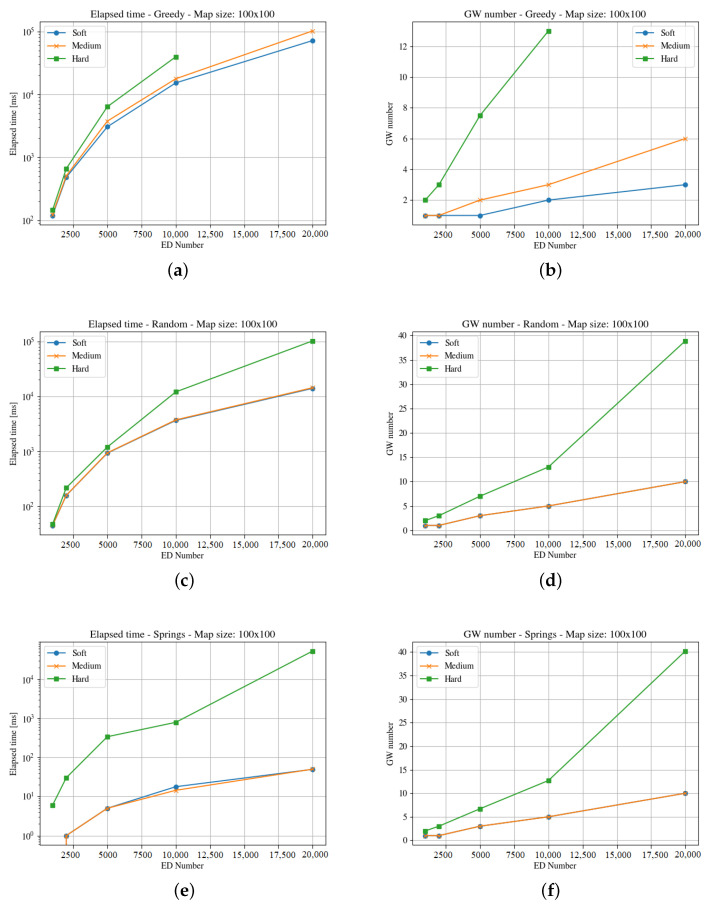
Results for the first map with uniform distribution. For the three GWs allocation methods, it considers time demanded and minimum number of gateways. (**a**) Greedy Elapsed. (**b**) Greedy Gateways. (**c**) Random Elpased. (**d**) Random Gateways. (**e**) Springs Elapsed. (**f**) Springs Gateways.

**Figure 5:**
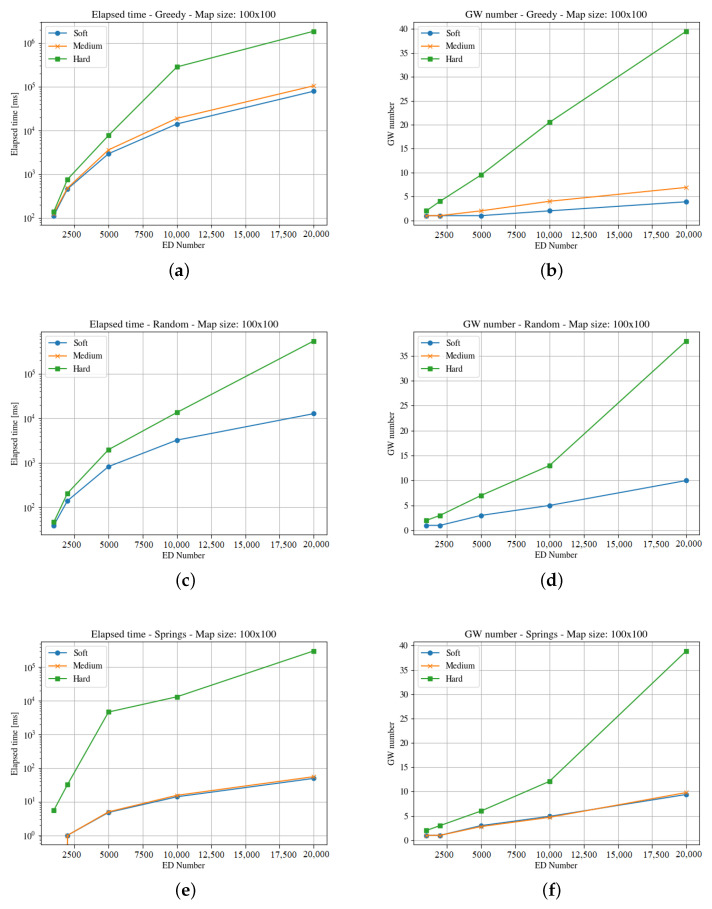
Results for the first map with cloud distribution. For the three GWs allocation methods, it considers time demanded and minimum number of gateways. (**a**) Greedy Elapsed. (**b**) Greedy Gateways. (**c**) Random Elapsed. (**d**) Random Gateways. (**e**) Springs Elapsed. (**f**) Springs Gateways.

**Figure 6:**
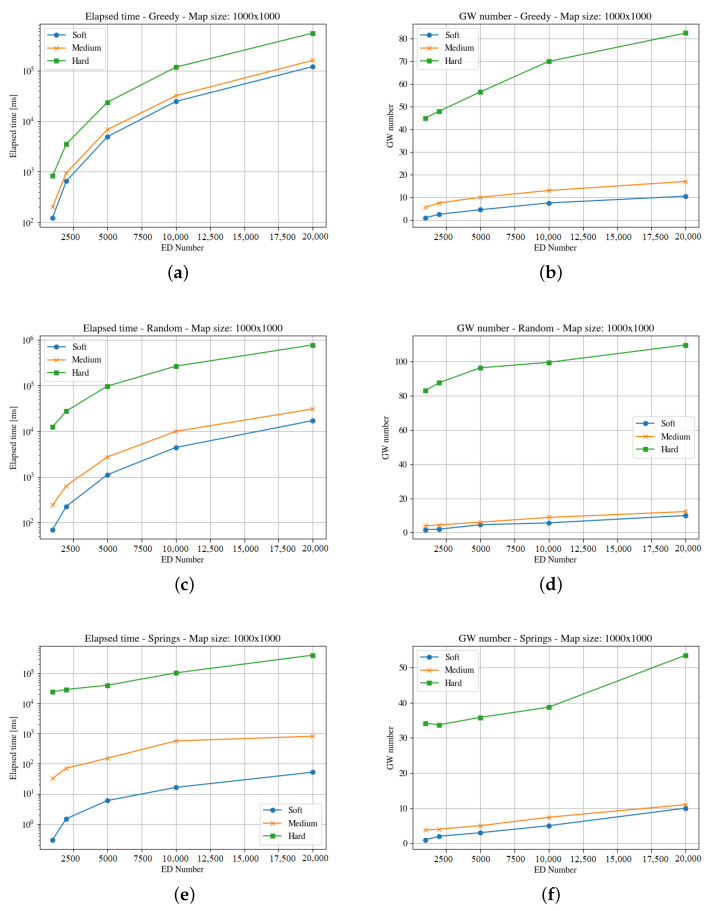
Results for the second map with uniform distribution. For the three GWs allocation methods, it considers time demanded and minimum number of gateways. (**a**) Greedy Elapsed. (**b**) Greedy Gateways. (**c**) Random Elapsed. (**d**) Random Gateways. (**e**) Springs Elapsed. (**f**) Springs Gateways.

**Figure 7:**
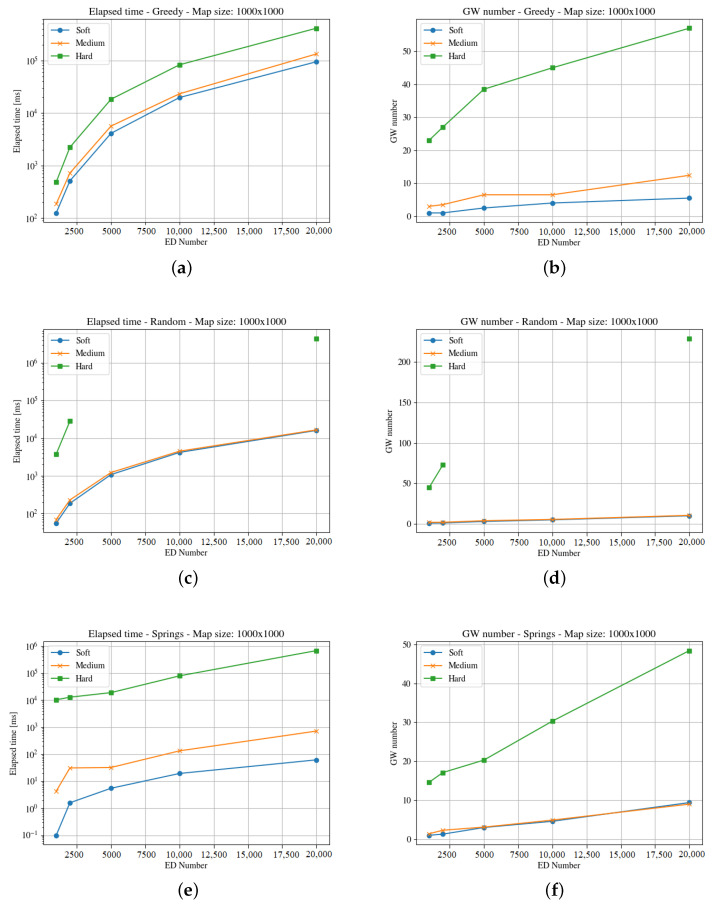
Results for the second map with cloud distribution. For the three GWs allocation methods, it considers time demanded and minimum number of gateways. (**a**) Greedy Elapsed. (**b**) Greedy Gateways. (**c**) Random Elapsed. (**d**) Random Gateways. (**e**) Springs Elapsed. (**f**) Springs Gateways.

**Figure 8:**
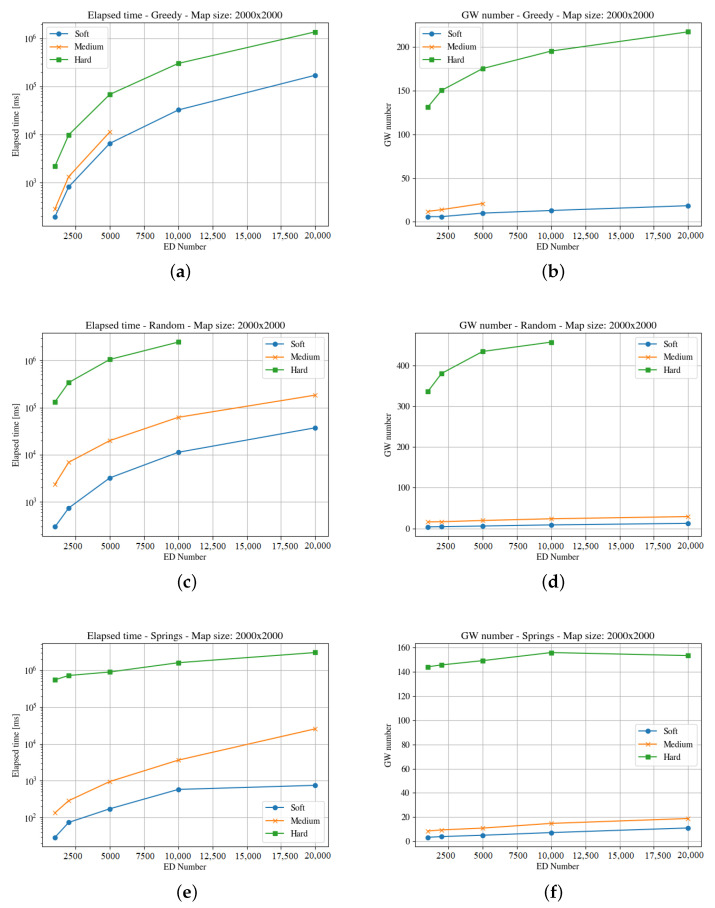
Results for the third map with uniform distribution. For the three GWs allocation methods, it considers time demanded and minimum number of gateways. (**a**) Greedy Elapsed. (**b**) Greedy Gateways. (**c**) Random Elapsed. (**d**) Random Gateways. (**e**) Springs Elapsed. (**f**) Springs Gateways.

**Figure 9:**
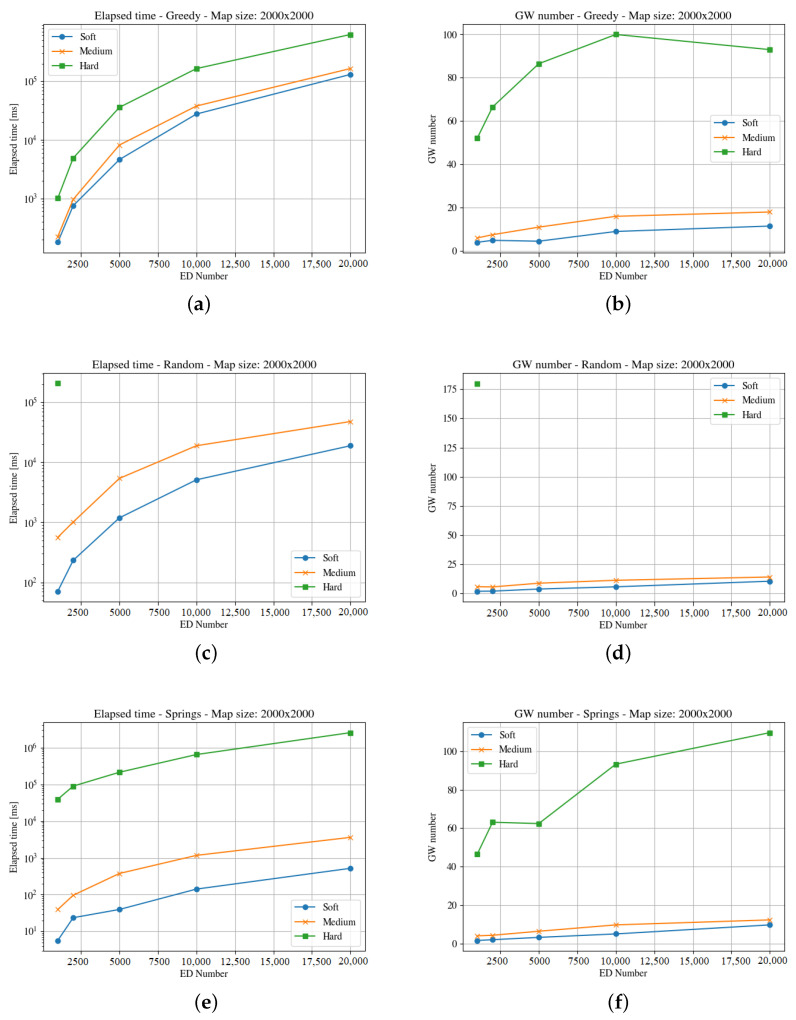
Results for the third map with cloud distribution. For the three GWs allocation methods, it considers time demanded and minimum number of gateways. (**a**) Greedy Elapsed. (**b**) Greedy Gateways. (**c**) Random Elapsed. (**d**) Random Gateways. (**e**) Springs Elapsed. (**f**) Springs Gateways.

Figure 4, Figure 5, Figure 6, Figure 7, Figure 8 and Figure 9 presents the simulation results for the three maps, both in time required to find an optimum solution and the number of gateways for the three time constraints types. In some figures the curves are not complete because there was a timeout while computing the solution, or the maximum number of iterations was reached not obtaining 100% coverage.

The first map is rather small. A square of 100 m is just a city block. In this scenario, a gateway placed in the middle of the square may listen to all the devices with all the SF. Figure 4 and Figure 5 show the results obtained for the computation time and gateways needed for both end-devices respectively, three optimization methods and three time constraints types.

The results show that the hard time constraints is always the one that needs two or three orders of magnitude (in terms of computation time) over soft time constraints, and also requires more gateways. Comparing both figures, it is clear that the clouds distribution is harder to solve.

It is important to note that even if a solution is found on time for 40 gateways, it is not feasible for the maximum number of end-devices, as the number of required channels is over 16. This is a serious restriction for using LoRaWAN in high density areas with hard time constraints.

The medium and soft time constraints are feasible, and solutions are find with all positioning approaches. It is interesting to note that the computation time for the Pseudo-Springs approach is significantly shorter, and the Greedy approach provides the minimum number of required gateways. This is a consequence of the allocation mechanism in such a small area.

The second map (i.e., the square of 1000 m) represents an area of one hundred blocks in a city. The heuristics solve the allocation problem deploying different numbers of gateways to cover the region. As can be seen in Figure 6 and Figure 7, the elapsed time to compute a solution for the hard time demand systems is like in the previous map; i.e., higher than the one needed for medium and soft cases, with both end-devices distribution.

The Spring methods obtains a better solution in much less time than the other methods, and clouds distributions demand more time than the uniform case. In this case, the raw data (https://github.com/matiasmicheletto/lorawan-simulation-results, accessed on 19 April 2023) shows that for the clouds distribution, the Greedy approach is unable to find a solution for the hard time demand systems in this map in half of the instances, for ten and twenty thousand end-devices. The situation is worst for the Random method, as it is not able to find a solution over two thousand end-devices. Timeout condition or a maximum iterations limit are reached, and the coverage is not complete so the results are not comparable with the rest of the methods.

The third map (i.e., the square of 2000 m) is four times the previous one. Figure 8 and Figure 9 present the results for the elapsed time and gateways required for both end-devices distributions. In this case, the random method is unable to find a solution within the time limit for the hard time constraints. A larger area implies lower end-devices density, and this forces more gateways to be deployed as the distance to cover is longer. Like in the previous cases, the Springs method provides the better results both considering the number of gateways and the time needed to compute the solution. In the case of the clouds distribution of end-devices, the Random method is unable to find a solution for any number of end-devices in the hard time constraint instances, and the greedy is unable to find a solution in the medium and hard time demand systems in half of the instances.

### 7.3. The Second Set of Experiments: Considerations and Results

In these simulations we kept constant the nodes density and used three map sizes with a uniform distribution of end-devices and a medium time constraint. The smallest map is a square of 500 m, with 2500 end-devices. In terms of size, the next map is a square of 1000 m, with 10 thousand end-devices. The largest map is a square of 2000 m, with forty thousand end-devices. This experiment was designed to evaluate the performance of the GWs positioning strategies, when they have to deal with a large area where thousand of end-devices are deployed.

In Table 3 a constant density is kept for three areas. As can be seen in the results, as the map increases, also the number of gateways and the time needed to compute the solution and the memory footprint demanded by the methods. What is interesting is that while the map size is increased four and sixteen times respectively, the number of required gateways increases also linearly, but the computation time increases exponentially.

In Table 3, the 500 m square area is considered the base and the results of the other maps are relative to the previous map size. In this way, the number of gateways for 500 m is unity (it is not the actual number, but the base), in the next map (that is four times larger) the number of gateways necessary to schedule all the end-devices, and also the time needed to compute this result, are relative to that previous case. As it can be seen, as the number of gateways increases proportional to the increase in the map size, the time needed to compute the result is incremented more than twenty times (500 m to 1000 m) and over fifty times (1000 m to 2000 m) for the other two areas.

The memory footprint is also an important fact to be considered for the implementation of this kind of systems. In this aspect, Greedy has a rather bad behavior, in comparison with the Random and Springs cases.

It is interesting to note that while the map size is quadruple, the ratio of gateways is slightly below four, indicating that optimizing the deployment in larger areas may have some benefit. However, the time demanded to compute a solution for the larger map is so much larger that the small average reduction is not significant.

## 8. Conclusions and Future Work

LoRaWAN has shown to be useful to support communication in IoT systems, although it is limited when real-time message delivery is required to support the systems operation. Therefore, using LoRaWAN is not always feasible when the communication involves time constraints. For economic and time reasons, that feasibility must be determined at early stages of any IoT system development project.

Today, there is not a simple way to determine such a feasibility at early stages of a project when the solution uses LoRaWAN as communication support. In this article we propose an Integer Linear Programming model to determine the feasibility of using this protocol in different settings and addressing several messages time constraints.

The model can use various heuristics to link end-devices to gateways, although in this article we have used only three approaches: greedy, random and pseudo-springs. The first one solves the nodes assignment by computing the end-devices with the greatest adjacency degree and transforming them in gateways. The second one uses a random deployment approach to position the gateways, and the third one, after an initial random deployment of gateways, moves these nodes towards the “center of mass” of the end-devices.

Two different experimental scenarios were set up to evaluate the gateways positioning approaches, in order to support time constraint messaging. In the first set of experiments, an extensive evaluation of the positioning approaches was made with 1700 instances, which allow us to perform a deep analysis of the different cases. The results show that the pseudo-spring approach is the only one that solves all the instances, obtaining the better results both in computation time and reaching the objective function (minimum number of gateways), while providing on-time scheduling to all the end-devices.

In the second set of experiments, we studied different alternatives to evaluate the performance of the heuristics to solve different map sizes with constant density of nodes. To address this assignment problem, the results show that it is convenient to partition the deployment area into smaller ones, as the number of gateways is not minimized further if larger spaces are considered. In these cases, the time needed to compute a solution increments exponentially with the size of the map and the number of end-devices to allocate and schedule. In fact, considering larger areas does not provide better solutions in relation to the number of required gateways or channels, and it demands two or three orders of more time to compute the results.

The allocation of end-devices to gateways was made only on the basis of minimizing the number of the latter. These are more expensive nodes that require better radio equipment, at least two types of network interfaces (one for LoRa devices and the other to connect into Internet), more memory to manage the two concurrent protocols, and an important CPU capability. However, depending on the type of the application on which the LoRaWAN network will finally operate, it may require also the minimization of the demanded energy or transmission active window. These other optimization objectives should be also supported by the model, and the gateways positioning heuristics should consider them in the search of a multi-objective optimization process. These aspects are part of the future work. 

## Figures and Tables

**Table 1:** Transmission bit rates and byte transmission delay.

*SF*	*BR* [b/s]	Delay per Byte [ms]
7	5468.8	1.5
8	3125.0	2.6
9	1757.8	4.6
10	976.6	8.2
11	537.1	14.9
12	292.9	27.3

**Table 2:** Symbol table.

Symbol	Meaning
*B*	Transmission banwidth used by the radio transducer.
*CR*	Code rate value related to the amount of redundancy bits used in the modulation.
*SF*	Spreading factor, it varies between 7 and 12.
*BR*	Bit-rate, number of bits per second transmitted by the radio transducer.
*Z*	Message length in bytes.
*c*	Message transmission time.
*T*	Message period.
*D*	Message deadline.
*ED*	End-device.
*GW*	Gateway.
*ch*	Channel.
*E*	Set of end-devices.
*G*	Set of gateways.

**Table 3:** Experiments involving a constant density of EDs.

Map Size	Greedy			Random			Springs		
	Rel *GW*	Rel Time	Mem [MB]	Rel *GW*	Rel Time	Mem [MB]	Rel *GW*	Rel Time	Mem [MB]
500	1	1	31.2	1	1	9.1	1	1	9.1
1000	3.8	29.8	389	4.4	36.3	54.2	3.6	40.5	54.2
2000	3.7	60.6	6114	4.5	52.8	600.7	4.1	136	600.7

## Data Availability

Simulation code and results are available at the following websites: https://github.com/matiasmicheletto/realtime-lorawan-simulator, accessed on 19 April 2023 http://www.ingelec.uns.edu.ar/rts/LoRaAllocSim, accessed on 19 April 2023. https://github.com/matiasmicheletto/lorawan-simulation-results, accessed on 19 April 2023.
